# Association of Physical Activity with Impulsivity, Depression and Anxiety Among Patients with Gambling Disorder: A Cross-Sectional Study

**DOI:** 10.3390/ijerph23050579

**Published:** 2026-04-29

**Authors:** Alicia Fernández-Parra, Juan Martín-Hernández, Azael J. Herrero, Inmaculada Fierro, Ana Domínguez-García, María Sol Cobo-Cuadrado, Pilar González-Pélaez, Carlos Roncero

**Affiliations:** 1 Department of Health Sciences, European University Miguel de Cervantes, 47012 Valladolid, Spain; jmartinh@uemc.es (J.M.-H.); jaherrero@uemc.es (A.J.H.); ifierro@uemc.es (I.F.); adominguez@uemc.es (A.D.-G.); mscobo@uemc.es (M.S.C.-C.); mpgonzalez@uemc.es (P.G.-P.); croncero@uemc.es (C.R.); 2 Psychiatric Area, Faculty of Medicine, University of Salamanca, 37007 Salamanca, Spain

**Keywords:** anxiety, behavioral addictions, depression, gambling disorder, impulsivity, physical activity

## Abstract

**Highlights:**

**Public health relevance—How does this work relate to a public health issue?**

- Gambling disorder (GD) represents a significant public health challenge due to its strong association with anxiety, depression, and impulsivity.
- This study evaluates physical activity as a potentially modifiable factor influencing mental health outcomes in individuals with GD.

**Public health significance—Why is this work of significance to public health?**

- High rates of anxiety and depression (~65%) confirm the substantial psychiatric burden in clinical GD populations.
- The absence of significant associations between physical activity and key symptoms questions its role as a standalone protective factor in GD.

**Public health implications—What are the key implications or messages for practitioners, policy makers and/or researchers in public health?**

- Interventions for GD should prioritize integrated, multidisciplinary strategies rather than relying solely on lifestyle approaches such as physical activity; however, from a public health perspective, physical activity should be systematically evaluated in these patients.
- Public health policies and future research should focus on comprehensive treatment models and on generating longitudinal evidence to more effectively address behavioral addictions.

**Abstract:**

Gambling disorder (GD) is a behavioral addiction associated with significant psychosocial consequences and high psychiatric comorbidity, including anxiety, depression, and impulsivity; however, the role of physical activity (PA) as a potential modulator of these alterations remains unclear. The aim of this study was to examine the relationship between PA levels and symptoms of anxiety, depression, and impulsivity in patients with GD. An observational study was conducted with 62 adults diagnosed according to *DSM-5* criteria, recruited from AJUPAREVA (Valladolid, Spain). PA was assessed using the International Physical Activity Questionnaire (IPAQ), Personality trails where evaluated with CEPER III, impulsivity with the Barratt Impulsiveness Scale (BIS-11) and the Plutchik Impulsivity Scale, and anxiety and depression with the Hamilton Anxiety (HAM-A) and Depression (HAM-D) scales. Participants were predominantly male (91.5%) and reported moderate-to-high PA levels. No significant differences were found in total impulsivity across PA levels; however, motor impulsivity was higher in highly active individuals, while non-planning impulsivity was greater in those with low PA. Anxiety and depression were highly prevalent (~65%) with no significant associations with PA. In conclusion, PA was not significantly associated with psychiatric outcomes in this clinical sample, highlighting the need for larger, longitudinal studies to clarify its potential role within multidisciplinary interventions for GD.

## 1. Introduction

Gambling disorder (GD) is classified as a behavioral addiction in the *Diagnostic and Statistical Manual of Mental Disorders* (5th ed., text rev.) [1,2] and is characterized by a persistent and recurrent pattern of gambling behavior that leads to clinically significant impairment or distress. Diagnosis requires the presence of at least four criteria within a 12-month period, with a particular emphasis on loss of control, persistence despite adverse consequences, and functional deterioration. The diagnostic framework prioritizes behavioral consequences over gambling frequency or financial losses, supporting the conceptualization of GD as a chronic addictive disorder. Its reclassification from an impulse control disorder to an addictive disorder reflects shared neurobiological mechanisms, including dysfunctions in reward-related circuitry, phenotypical similarities with substance use disorders, and comparable treatment responses [3,4].

GD is associated with substantial psychosocial and clinical burden, including financial difficulties, occupational impairment, interpersonal problems and domestic violence [5,6,7,8,9]. Psychiatric comorbidity is highly prevalent, particularly with mood, anxiety, and substance use disorders, and suicidal ideation and attempts are common among treatment-seeking individuals [4,10,11,12,13].

Impulsivity has been consistently identified as a core vulnerability factor in GD [1,14]. Evidence suggests that impulsivity functions largely as a stable personality trait [15] and is associated with greater disorder severity, partly through its relationship with anxiety and depressive symptoms [12]. Neurocognitive studies have documented impairments in inhibitory control, temporal discounting, and decision-making [16], and distinct clinical subtypes have been described based on impulsivity and emotional dysregulation profiles [11]. Specific impulsivity dimensions, such as urgency and lack of premeditation, have been linked to indebtedness, cognitive distortions, relapse, and treatment dropout [17,18].

In parallel, increasing attention has been given to the role of physical activity (PA) in addictive and affective disorders [4,19]. In GD, structured PA interventions have been associated with reductions in anxiety and gambling craving, potentially mediated by neuroendocrine mechanisms [4]. However, findings remain inconsistent. Higher gambling prevalence has been observed in highly sport-involved populations [19], and positive associations between PA levels and other behavioral addictions have been reported [20]. Moreover, personality traits appear to influence PA engagement in individuals with GD [21].

Despite robust evidence linking impulsivity and emotional distress to GD severity, the potential regulatory role of physical activity within this framework remains insufficiently understood. In particular, limited research has examined how PA levels interact with anxiety, depression, and impulsivity in clinical GD samples. Therefore, the aim of the study is to investigate the relationship between physical activity, anxiety, depression, and impulsivity in patients with GD.

## 2. Materials and Methods

### 2.1. Study Design and Participants

An observational study was conducted in patients diagnosed with gambling disorder (GD) according to the *DSM-5* criteria [2]. Inclusion criteria were adults (≥18 years) with a GD diagnosis recruited from the Association of Players in Rehabilitation (AJUPAREVA, Valladolid, Spain).

**Figure 1:**
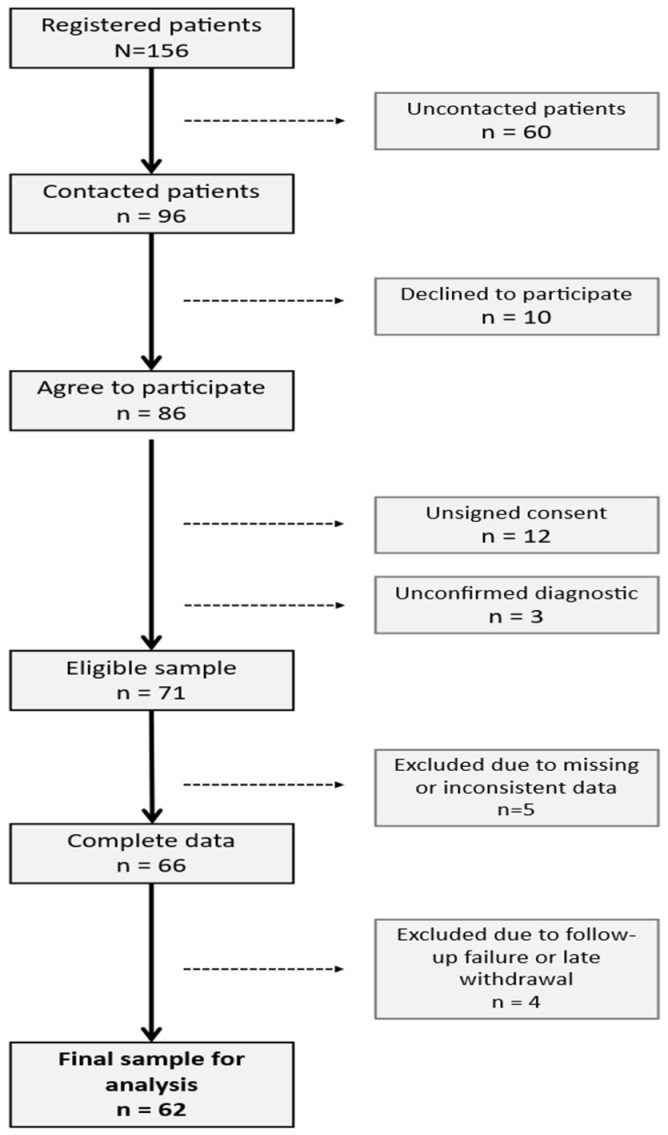
Flowchart for the inclusion of patients in the study.

All 156 patients registered at AJUPAREVA were invited to participate in the study via email, telephone, or in person. Of these, 96 responded to the invitation, and 10 declined to participate. Among the 86 individuals who agreed to take part, 12 were excluded due to failure to provide informed consent and 3 due to an unconfirmed diagnosis of gambling disorder. Consequently, the final eligible sample consisted of 71 patients (Figure 1). Subsequently, 5 participants were excluded due to missing or inconsistent data, leaving a dataset of 66 patients with complete data. Finally, 4 additional participants were excluded due to follow-up failure or late withdrawal, resulting in a final sample for analysis of 62 participants.

After agreeing to participate, all participants completed the CEPER-III [22] and IPAQ [23] questionnaires. Sociodemographic variables and clinical history were collected from the participants’ admission interviews at the association and incorporated into a database. Clinical diagnoses were established by a trained psychiatric in accordance with *DSM-5* criteria through a structured clinical interview. Questionnaires and assessment scales were administered for this research by the first author (AFP), a trained psychologist. The results of the questionnaires were added to this database. Both questionnaires were administered by a collaborating psychologist from AJUPAREVA, and the researchers received an anonymized Excel database for analysis.

In addition to the core variables of the study (personality traits and level of physical activity), other variables were taken into account. These were obtained from patients’ responses during the admission interview at the association, as previously indicated. The variables included: sex, age, marital status, educational level, employment status, type of gambling dependence, episodes of consumption, presence of withdrawal syndrome, time elapsed from when the patient became aware of their gambling problem to seeking help at the center, estimated total losses, family financial situation, academic difficulties, and mental health history.

CEPER-III (Cuestionario Exploratorio de la Personalidad-III) [22,24] (The Exploratory Personality Questionnaire-III (CEPER-III)) is a psychometric instrument designed to assess a wide range of personality traits in adults in an exploratory manner. It covers multiple dimensions such as anxiety, impulsivity, and sociability, providing a detailed individual profile, and has been widely used to study this population. Internal consistency of the scales was assessed using Cronbach’s alpha, which ranged from 0.75 to 0.89, with an overall alpha for the test of 0.97 [22].

With regard to the International Physical Activity Questionnaire (IPAQ) [23] researchers developed several versions of the instrument according to the number of questions (short or long), the recall period (“usually in 1 week” or “last 7 days”) and the method of application (self-administered survey, face-to-face interview or by telephone). The questionnaires were designed to be used in adults aged 18 to 65 years. We used the short version, which consists of 9 items and provides information on the time spent walking, in moderate- and vigorous-intensity activities and in sedentary activities. Different studies suggest that the short version is the one used in population studies [25,26]. We analyzed the IPAQ levels in their original three categories: low, moderate, and high. The IPAQ-E physical activity questionnaire is a reliable and valid instrument for the assessment of physical activity in Spanish-speaking contexts. The Spanish version of the International Physical Activity Questionnaire (IPAQ), both the short and long forms, demonstrates reasonable psychometric properties and good reliability for assessing physical activity levels in adults. It shows acceptable agreement between versions and good reliability, as assessed by Spearman’s correlation coefficient, commonly around 0.8 (*r* = 0.81; 95% CI: 0.79–0.82 for the long version) [27].

To evaluate impulsiveness, we used the Barratt Impulsiveness Scale, 11th version (BIS-11) [28]. The BIS-11 is a self-report questionnaire, which contains 30 questions that need to be scored on a scale from 1 to 4 (1 = rarely/never; 2 = occasionally; 3 = often; 4 = almost always/always). Factor analysis includes 6 first-order factors (attention, motor impulsiveness, self-control, cognitive complexity, perseverance and cognitive instability) and three second-order factors (attentional impulsiveness, motor impulsiveness and non-planning impulsiveness). The BIS-11 total score indicates the level of impulsiveness. The higher the BIS-11 total score, the higher the impulsiveness level is. The questionnaire contains statements that indicate impulsive behavior (“I do things without thinking”) and statements that indicate non-impulsive behavior (“I am self-controlled”). The BIS-11 is the most frequently used self-report measure of impulsivity. It has been adapted for use in Spanish populations by Oquendo and cols [29]. The scale is self-administered. In the Spanish validation, linguistic equivalence, conceptual equivalence, and scale equivalence were satisfactory. The proportion of agreement between the English and Spanish versions ranges from 0.67 to 0.80. Additionally, internal consistency is high, with values around 0.8 test–retest reliability over a 2-month period.

The Plutchik impulsivity scale is a clinician-administered instrument for evaluation impulsivity consisting of items [30]. It was validated by Rubio and cols. in 1998 [31]. The scale has demonstrated good concurrent validity and good reliability (Internal Consistency): moderate to good Cronbach’s alpha values were obtained (α = 0.73) in the Spanish adaptation.

The Spanish version of the Hamilton Anxiety Rating Scale (HAM-A) was used to assess the severity of anxiety symptoms. This clinician-administered instrument, developed by Hamilton (1959) [32], is designed for use in adult populations. The scale consists of 14 items evaluating both psychic and somatic components of anxiety, including anxious mood, tension, fears, sleep disturbances, and autonomic symptoms. Each item is scored on a 5-point Likert scale ranging from 0 (not present) to 4 (very severe), yielding a total score between 0 and 56, with higher scores indicating greater anxiety severity. Based on commonly used cut-off points, anxiety levels were classified as mild (≤17), moderate (18–24), and severe (≥25). The HAM-A has demonstrated adequate psychometric properties and is widely used in clinical and epidemiological studies to assess anxiety severity and monitor symptom changes over time. The Spanish version was validated by Lobo and cols. (2002) [33]. The Psychometric Properties of this version were internal consistency (Cronbach’s; HARS = 0.89), test–retest reliability, and inter-rater reliability (intraclass correlation coefficient: HARS = 0.92 and 0.92, respectively). Additionally, the Spanish version of the Hamilton Depression Rating Scale (HAM-D) [34] was used to assess the severity of depressive symptoms. This clinician-administered instrument, developed by Hamilton (1960) [34], is designed for use in adult populations. The scale consists of 17 items (classic version) evaluating affective, cognitive, behavioral, and somatic symptoms of depression, including depressed mood, anxiety, sleep disturbances, somatic complaints, and suicidal ideation. Each item is scored on a 0–2 or 0–4 scale, depending on the item, yielding a total score between 0 and 52, with higher scores indicating greater depression severity. Based on conventional cut-off points, depression levels were classified as mild (≤7), moderate (8–16), and severe (≥17). The HAM-D has demonstrated adequate psychometric properties and is widely used in clinical and epidemiological studies to assess depression severity and monitor symptom changes over time. The Spanish version was validated by Conde and cols. (1974) [35]. The scale demonstrates good reliability, with adequate internal consistency (Cronbach’s α ranging from 0.76–2). Regarding validity, the scale shows high correlations with other instruments for the assessment of depression (*r* = 0.8).

### 2.2. Statistical Analysis

Descriptive statistics were calculated as frequencies and percentages for categorical variables and as mean ± standard deviation (SD) for continuous variables. The distribution of continuous variables was assessed using the Shapiro–Wilk test. Because several variables did not meet normality assumptions and the sample was small and unbalanced across physical activity categories, non-parametric tests were applied. Kruskal–Wallis tests were used to compare impulsivity scores (BIS-11 subscales, BIS-11 total score, and Plutchik score) across the three IPAQ categories (low, moderate, and high. Associations between IPAQ categories and dichotomized anxiety/depression status were examined using exact tests for categorical data; Fisher’s exact test was used for 2 × 2 comparisons, and the exact test for r × c tables was used when expected cell frequencies were small. All tests were two-tailed, and statistical significance was set at *p* < 0.05. Analyses were performed using IBM SPSS Statistics, version 28.0.

### 2.3. Ethical Considerations

This study was performed in accordance with the Declaration of Helsinki on medical research in human beings in its latest version and with the applicable regulations on Good Clinical Practice. The confidentiality of the participants’ personal data was preserved in accordance with Organic Law 3/2018, of 5 December, on the Protection of Personal Data and guarantee of digital rights. Informed consent was obtained from each participant. The studies involving humans were approved by Research Ethics Committee of the European University Miguel de Cervantes, Valladolid (Spain) (Reference: 8/2023). The studies were conducted in accordance with the local legislation and institutional requirements.

## 3. Results

The sociodemographic characteristics and physical activity levels of the 62 pathological gamblers (mean age 40, median 41 and SD 11) included in the study are described in Table 1.

The study has clinical particularities because 48.5% of the sample were Dual Disorders patients and 48.5% presented an SUD. Differences in this characteristic were not observed according to the status of rehabilitation or non-rehabilitation (they have been not gambling more than 2 years).

Regarding personality trails compared with the Spanish general population according to CEPER-III, differences were detected among men in paranoic, histrionic, narcissistic and sadistic traits, and among women in histrionic and narcissistic traits [21]. Due to this, we can confirm that it is not a general population.

The level of impulsivity in the sample was high. Total scores and subcomponents of impulsivity (cognitive, motor, and non-planned) were compared across low, moderate, and high physical activity groups (Table 2). The results show that impulsivity scores did not differ substantially according to the level of physical activity. In the BIS-11, total impulsivity was similar across groups (low: 54.00 ± 11.51; moderate: 52.81 ± 12.20; high: 56.58 ± 14.52). However, motor impulsivity was higher in the high-activity group (18.60 ± 7.14) compared to the low (13.80 ± 7.60) and moderate (15.27 ± 6.28) groups, whereas non-planned impulsivity was greater in the low-activity group (24.50 ± 4.01) relative to moderate (22.88 ± 6.81) and high (21.52 ± 7.08) levels. Plutchik scores remained practically constant across physical activity levels (17.56–18.32). Overall, the data suggest no statistically significant differences in global impulsivity between groups.

Table 3 and Table 4 examine the association between physical activity level and the presence of anxiety and depression symptoms, assessed with the HAM-A and HAM-D scales.

## 4. Discussion

The present study examined the associations between physical activity (PA), impulsivity, anxiety, and depression in a clinical sample of patients with gambling disorder (GD). Overall, the findings reinforce the conceptualization of GD as a behavioral addiction characterized by high emotional distress and pronounced impulsivity, while questioning the role of habitual physical activity as a straightforward protective factor in this population.

Consistent with previous research, impulsivity levels were elevated across the sample, supporting its role as a core and relatively stable vulnerability factor in GD [16,36]. Both meta-analytic and clinical evidence indicate that impulsivity in GD is not merely situational but reflects enduring personality-related dysregulation linked to impaired inhibitory control, decision-making, and emotional regulation [11,16,17,37]. In our study, total impulsivity scores did not differ significantly across PA levels, suggesting that global impulsive traits are not substantially modulated by self-reported activity engagement. This aligns with findings indicating that impulsivity in GD frequently persists despite changes in lifestyle behaviors or behavioral activation [36].

However, the observed differences in impulsivity subdimensions deserve attention. Higher motor impulsivity among highly active individuals and greater non-planning impulsivity in those with low PA suggest that PA may interact selectively with specific behavioral expressions of impulsivity rather than with impulsivity as a unitary construct. Motor impulsivity, characterized by action without forethought, has been linked to heightened arousal and sensation-seeking tendencies in GD [14,16], which may partly explain its elevation among individuals engaging in high PA. Conversely, non-planning impulsivity, reflecting limited future orientation, may be more prominent in sedentary individuals, potentially mirroring broader self-regulation difficulties [18]. These findings support the relevance of differentiating impulsivity dimensions when examining behavioral correlates in GD populations [11].

Anxiety and depressive symptoms were highly prevalent across all PA levels, with approximately two-thirds of participants presenting clinically relevant symptomatology. This is consistent with the extensive literature documenting high rates of affective and anxiety disorders in individuals with GD [7,38]. Importantly, no significant associations were detected between PA level and either anxiety or depression severity. Although factors as psychiatry or medical comorbidity including weigh or weight stigma could play a role on the relationship [39,40], our results contrast with evidence from intervention studies suggesting that structured exercise programs can reduce anxiety and emotional distress in GD when integrated into treatment settings [3,4,10,41]. The discrepancy may indicate that unstructured or self-directed PA does not yield the same psychological benefits as supervised, therapeutically framed exercise interventions.

From a behavioral addiction perspective, high PA should not automatically be interpreted as protective. Previous studies have reported increased gambling prevalence and severity among individuals with high sport or fitness involvement, suggesting that elevated PA may coexist with, or even reflect, heightened arousal-seeking or compulsive tendencies [19]. Moreover, research on other behavioral addictions has shown that PA can function as an avoidance strategy or as part of maladaptive coping patterns rather than as an effective regulator of emotional distress [20]. Within this framework, the lack of association between PA and reduced anxiety or depression in the present study suggests that PA may operate as a neutral or context-dependent behavior in GD rather than a consistently beneficial one.

Taken together, these findings underscore the complexity of the relationship between PA and mental health outcomes in GD [42,43]. While PA is widely promoted as a positive health behavior, its psychological impact in populations characterized by impulsivity and emotional dysregulation appears to be neither linear nor uniform. Clinical and rehabilitative evidence supports the utility of PA when embedded within structured, multidisciplinary treatment programs for GD [4,10,13,39], but the present results caution against assuming that higher habitual PA alone is sufficient to alleviate core psychopathological features of the disorder.

### Limitations

The study has several limitations. Given the cross-sectional design and the sample size was small and unbalanced, particularly in the low physical activity group, which reduces statistical power and generalizability. Its cross-sectional design prevents causal inferences between physical activity, impulsivity, anxiety, or depression. Data were self-reported, potentially introducing recall or social desirability bias. Confounding factors such as substance use, stress, medical conditions, gambling type, sleep, psychiatric history, and socioeconomic status were not controlled. Anxiety and depression were measured at a single time point, limiting interpretation of symptom trajectory. So, conclusions must be interpreted cautiously, and the findings may not be generalizable beyond pathological gamblers. Additionally, we did not study the gender aspect due to the low number of women. Previously, it has been described that gambling is markedly more common among males than among females (12 vs. 1 percent) [19]. Additionally, reliance on self-report measures introduces potential recall or social desirability biases, particularly concerning PA and impulsivity.

We detected that GD is characterized by elevated impulsivity and broad emotional distress, and that physical activity does not moderate these relationships in simple or linear ways. Consistent with initial guidance, PA among individuals with GD should be carefully monitored, ideally maintained at moderate levels, and embedded within structured treatment to avoid maladaptive overuse. High levels of exercise may constitute a marker of anxiety and are associated with gambling behavior. Physical activity may function as a mediating variable. Although this effect was not observed in our study, previous research has described an inverted U-shaped relationship. Consequently, physical activity in individuals with GD should be maintained at moderate levels [4]: while physical activity may contribute to improvements in anxiety and depressive symptoms, it should be carefully regulated and monitored. Nowadays, the approach to behavioral addictions presents multiple questions [42,43].

## 5. Conclusions

From a public health perspective, physical activity represents a relevant behavior to consider in populations with gambling disorder (GD). The results highlight the complexity of GD and the multifactorial interactions among impulsivity, affective symptoms, and anxiety. Although physical activity is widely recognized as beneficial for general health, its specific role in relation to psychiatric symptoms within severely affected clinical populations such as individuals with GD remains uncertain. These observations underscore the relevance of evaluating PA adopting cautious, individualized, and multidimensional approaches.

PA should be considered a complementary, carefully regulated component of integrated treatment approaches rather than as a standalone strategy. Future studies employing longitudinal designs and larger samples are warranted to clarify potential causal relationships and to determine whether variations in physical activity are meaningfully associated with changes in impulsivity, affective, or anxiety symptoms over time, including the possibility of nonlinear effects of PA.

## Figures and Tables

**Table 1:** Sociodemographic characteristics and level of physical activity.

Variable	Category	Frequency (*n*)	Percentage (%)
Sex	Female	5	8.1
	Male	57	91.9
Age (years)	20–30	16	25.8
	31–40	15	24.2
	41–50	17	27.4
	>50	14	22.6
Physical activity level (IPAQ)	Low	10	16.1
	Moderate	27	43.5
	High	25	40.3
Total		62	100

Note: Values are expressed as absolute frequencies and percentages. IPAQ = International Physical Activity Questionnaire.

**Table 2:** The relationship between impulsivity and physical activity level, assessed using the BIS-11 (Barratt) and the Plutchik scale.

Physical Activity Level	N	Barratt Cognitive (M ± SD)	Barratt Motor (M ± SD)	Barratt Non-Planning (M ± SD)	Barratt Total (M ± SD)	Plutchik (M ± SD)
Low	10	15.70 ± 3.20	13.80 ± 7.60	24.50± 4.01	54.00 ± 11.51	17.89 ± 6.72
Moderate	26	14.65 ± 4.33	15.27 ± 6.28	22.88± 6.81	52.81 ± 12.20	17.56 ± 4.43
High	25	15.83 ± 4.69	18.60 ± 7.14	21.52± 7.08	56.58 ± 14.52	18.32 ± 6.18
Total	61	15.30 ± 4.29	16.39 ± 7.01	22.59± 6.55	54.52 ± 12.98	17.92 ± 5.47

Note: Data are presented as M ± SD = mean ± standard deviation; N indicates the sample size, and the *p*-value corresponds to comparisons of impulsivity (Barratt and Plutchik) across levels of physical activity. We used the Kruskal–Wallis statistic: IPAQ-Barratt *p* 0.54 and the IPAQ Plutchick *p* 0.68.

**Table 3:** Cross-tabulation between physical activity level (IPAQ) and depression (HAM-D).

IPAQ	Depression Light (*n*)	% Within IPAQ	Depression Moderate (*n*)	% Within IPAQ	Depression Severe (*n*)	% Within IPAQ	Depression Total (*n*)	% Within IPAQ
Low	3	30.0%	3	30.0%	4	40.0%	10	100.0%
Moderate	11	40.7%	7	25.9%	9	33.3%	27	100.0%
High	7	28.0%	10	40.0%	8	32.0%	25	100.0%
Total	21	33.9%	20	32.3%	21	33.9%	62	100.0%

Note: Fisher’s exact test (two-tailed) *p* = 0.806 > 0.05. There is insufficient evidence to conclude that the variables are associated; they are considered independent.

**Table 4:** Cross-tabulation between physical activity level (IPAQ) and anxiety (HAM-A).

IPAQ	Anxiety Light (*n*)	% Within IPAQ	Anxiety Moderate (*n*)	% Within IPAQ	Anxiety Severe (n)	% Within IPAQ	Anxiety Total (*n*)	% Within IPAQ
Low	6	60.0%	2	20.0%	2	20.0%	10	100.0%
Moderate	18	66.7%	3	11.1%	6	22.2%	27	100.0%
High	13	52.0%	2	8.0%	10	40.0%	25	100.0%
Total	37	59.7%	7	11.3%	18	29.0%	62	100.0%

Note: Fisher’s exact test (two-tailed) *p* = 0.530 > 0.05. There is insufficient evidence to conclude that the variables are associated; they are considered independent.

## Data Availability

The data used in this article are not readily available, as the main information from the study is included in the article. For any additional inquiries or requests to access the datasets, please contact the corresponding author at afernandezp@uemc.es.

## Abbreviations

AJUPAREVA	Asociación de Jugadores en Rehabilitación (Valladolid, España)
APA	American Psychiatric Association
BIS-11	Barratt Impulsiveness Scale, 11th version
CEPER-III	Caballo Personality Assessment Questionnaire (Cuestionario de Evaluación de Personalidad de Caballo)
*DSM-5*/*DSM-5-TR*	*Diagnostic and Statistical Manual of Mental Disorders, 5th edition, text revision*
GD	Gambling Disorder
HAM-A	Hamilton Anxiety Rating Scale
HAM-D	Hamilton Depression Rating Scale
IPAQ	International Physical Activity Questionnaire
M	Mean
PA	Physical Activity
SD	Standard Deviation
SPSS	Statistical Package for the Social Sciences
